# Long-term Developmental Trends of Pediatric Mitochondrial Diseases: The Five Stages of Developmental Decline

**DOI:** 10.3389/fneur.2017.00208

**Published:** 2017-05-17

**Authors:** Soyong Eom, Young-Mock Lee

**Affiliations:** ^1^Epilepsy Research Institute, Yonsei University College of Medicine, Seoul, Korea (Republic of); ^2^Department of Pediatrics, Gangnam Severance Hospital, Yonsei University College of Medicine, Seoul, Korea (Republic of)

**Keywords:** mitochondrial disease, developmental quotient, metabolic disease, pediatric epilepsy, brain atrophy

## Abstract

Mitochondrial diseases (MDs) are a heterogeneous group of progressive multisystem disorders caused by impaired mitochondrial function. This study aimed to evaluate the clinical course and long-term development of 53 pediatric patients with MDs. Developmental function was evaluated at nine time points (two pre-diagnosis, one at diagnosis, and six post-diagnosis), with the developmental quotient (DQ) from the Korean infant and child development test (KICDT) assessing a child’s developmental age (rather than chronological age). Additionally, disease-related clinical variables were reviewed, and clinical progress was determined through observation. Subgroup analyses by epilepsy severity, syndromic diagnosis, diffuse brain atrophy, and clinical rating were performed. The pre- and post-diagnosis results were compared by the paired *t*-test and Bonferroni correction. The pre-diagnostic, diagnostic, and post-diagnostic evaluations were compared using repeated measures ANOVA. Patients with diffuse brain atrophy at the first pre-diagnostic and second post-diagnostic evaluations showed lower DQs. Compared with patients with a mildly or severely deteriorating clinical course, those with an improving or static clinical course presented higher DQs at the pre-diagnostic and diagnostic evaluations. The age at onset of the first symptom correlated positively with the DQ post-diagnosis. Follow-up revealed consistent patterns of significant developmental deterioration during the lead time to diagnosis, with no significant decline post-diagnosis. The DQ is a feasible predictor and a measure of long-term functional development in children with MD. Early initiation of treatment may minimize developmental regression.

## Introduction

Mitochondrial diseases (MDs) vary considerably in terms of their manifestations and effects on different organ systems, as well as in terms of age of onset and rate of progression (1). Clinical pediatric reports have described the spectrum of signs and symptoms associated with MDs. Classification relies on accurate clinical, biochemical, and genetic information and may be based on either genotype or phenotype, but there is significant overlap. Although the prognosis for MD is dependent on the underlying diagnosis, there is large phenotypic variation (2, 3). Thus, the diagnostic evaluation is necessarily multitiered and broad based, with a focus on integrating information from many avenues (4). In particular, children with encephalopathy experience many limitations in daily life (5). However, information on the long-term clinical course and developmental outcome of this heterogeneous group of diseases is lacking (5–7).

One of the key needs of families caring for children with rare metabolic or genetic impairments is to understand the long-term effect of the disorder on the child’s development (8). Specifically, a better understanding of the needs, problems, and long-term developmental trends of these children is essential for effective care planning and improving the quality of life (1).

Accordingly, the aims of this study were to evaluate the clinical course and long-term developmental trends in pediatric patients with MDs. The present study is the first to attempt to use a developmental scale for retrospectively assessing and describing the long-term developmental trends of children with MDs.

## Materials and Methods

### Patients and Procedures

Data regarding pediatric patients were obtained from hospital records. Patient selection was based on both the diagnosis of MD and the results of a developmental evaluation using the developmental quotient (DQ). All patients were diagnosed with mitochondrial respiratory chain complex defects by biochemical enzyme assay of muscle tissue samples and satisfied the modified MD criteria proposed by Bernier et al. (9).

Standardized evaluations of developmental function were conducted. In addition, disease-related clinical variables were examined, including age at symptom onset, age at diagnosis, lead time to diagnosis, type of first symptom, lactic acidosis, pathological features indicating myopathies, result of the biochemical enzyme assay for the mitochondrial respiratory chain complex, neuroimaging findings, and syndromic diagnosis.

Lead time to diagnosis was defined as the time between first symptom onset and diagnosis of MD. The severity of serum lactic acidosis was categorized as mild, moderate, or severe, based on the increase over normal reference values (≥2-fold, ≥3-fold, or ≥4-fold increase). Normal reference range of lactate is known as 0.5–2.2 mmol/L (4). Myopathies were classified depending on whether abnormal pathology was detected only on one type of microscopy (one pathology) or on both light and electron microscopies (two pathologies). The diagnoses of mitochondrial encephalomyopathy, lactic acidosis, and stroke-like episodes (MELAS) and Leigh disease were based on the diagnostic criteria reported by Yatsuga et al. (10) and Rahman et al. (11), respectively. Involvement of the central nervous system, either with or without the involvement of other organs, was taken into consideration in assessing the severity of organ involvement in MD. The interpretation of patients’ diffuse brain atrophy on MRIs by the radiologist was reviewed, and the results were classified by three levels as no atrophy, mild atrophy, and severe atrophy by the pediatric neurologist.

The patients were divided into three groups based on the presence, severity, and intractability of epilepsy: patients without epilepsy; patients with drug-responsive epilepsy, defined as an epileptic condition responsive to antiepileptic treatment; and patients with drug-resistant epilepsy, requiring more than two tolerated and appropriately chosen and used antiepileptic drug schedules (12). Clinical progress was classified as improving, static, mildly deteriorating, or severely deteriorating, based on the changes in the clinical features of the patient, as observed by the treating clinician (modified from a previously published study) (13).

After the diagnosis of MD was confirmed, all patients were treated with mitochondrial cocktails such as coenzyme Q, carnitine, and various vitamins (14). All procedures conducted were approved by the institutional review board of Gangnam Severance Hospital in Seoul, Korea.

### Measures of Developmental Function

Developmental function was evaluated using the Korean infant and child development test (KICDT). This test was developed by the Development Evaluation Enacting Subcommittee of the Korea Pediatrics Academy in February 2002. The KICDT uses a development index to assess the development of toddlers younger than 5 years of age, whose development level is below the expected based on their chronological age in months. The KICDT can be used to quickly screen toddlers who are suspected of having delayed development or to observe toddlers who are already receiving treatment after positive diagnosis of delayed development (15). The KICDT is a 140-item, clinician-rated inventory of skills designed to assess a child’s developmental age (rather than chronological age) in five functional domains: gross motor, fine motor, social-personal, language, and cognitive-adaptive skills.

The DQ was calculated for each KICDT domain using the following formula: DQ = (developmental age/chronological age) × 100. The mean of the five domain DQs was designated the overall DQ and was used as the primary index of the child’s overall developmental level. An overall DQ below 80 was regarded as abnormal.

The attending pediatricians administered the KICDT and obtained the DQ scores for a total of up to nine evaluations. Pre-diagnostic developmental evaluations were performed twice (the first visit prior to diagnosis, pre-first and the second visit prior to diagnosis, pre-second), followed by a one-time diagnostic evaluation and six post-diagnostic developmental evaluations (the first visit after diagnosis, post-first; the second visit after diagnosis, post-second; the third visit after diagnosis, post-third; the fourth visit after diagnosis, post-fourth; the fifth visit after diagnosis, post-fifth; and the sixth visit after diagnosis, post-sixth). To analyze the overall developmental trends, we included the results of the developmental evaluations at all nine time points. For further analyses (patient subgroups and DQ subdomains), only five DQ values at the following time points were used: two pre-diagnostic evaluations (pre-first and pre-second), one-time evaluation during the diagnosis, and two post-diagnostic evaluations (post-first and post-second).

### Statistical Analysis

Univariate (mean, SD, range) and multivariate (Pearson correlation) descriptive statistics were used to analyze patients’ characteristics and clinical variables. In addition, Student’s *t*-test and the Mann–Whitney *U*-test (for non-parametric ordinal data) were performed to compare the subgroups in terms of epilepsy severity, syndromic diagnosis, diffuse brain atrophy, and clinical rating. The paired *t*-test with Bonferroni correction was used to compare the results of the developmental function evaluations (overall DQ and DQ for each KICDT domain) at pre- and post-diagnostic visits for various subgroups of patients and in the total cohort. Repeated measures ANOVA was performed to compare the evaluation results during the pre-diagnostic, diagnostic, and post-diagnostic visits. A *post hoc* test with Bonferroni adjustment was also conducted. IBM SPSS version 20.0 (IBM, Armonk, NY, USA) was used for data processing and analysis.

## Results

### Patient Characteristics

Our study included 53 children diagnosed with MD, whose developmental function had been assessed at several time points (pre-diagnosis, at diagnosis, and post-diagnosis) between March 2006 and February 2015. The lead time to diagnosis was 1.09 ± 1.15 years (range, 0.6–1.46 years), and the follow-up duration from pre-first to post-second was 5.45 years.

Forty patients (76%) had been diagnosed with non-specific MD, 11 (21%) with Leigh syndrome, and 2 (4%) with MELAS. The mean age at the first onset of symptoms was 1.0 ± 1.1 years (range, 0–4.2 years), and 32 (60%) of the children were boys. The most common first symptom was seizure (26, 49%), followed by delayed development (22, 42%).

The majority of patients showed static (20, 38%) or mildly deteriorating (22, 42%) clinical progress, whereas the rest exhibited severely deteriorating (10, 19%) or improving (1, 2%) clinical progress. More details of the clinical characteristics are presented in Tables 1 and 2.

**Table 1:** **Diagnostic evaluation of children with MD (*n* = 53)**.

Characteristic		Value
Male sex		32 (60)
Age at first symptom onset, years		0.95 ± 1.07
Age at diagnosis, years		3.12 ± 2.49
Lead time to diagnosis, years		2.17 ± 2.11
First symptom	Seizure	26 (49)
	Delayed development	22 (42)
	Visual disturbance	1 (2)
	Ataxia	1 (2)
	Dystonia	1 (2)
	Nystagmus	1 (2)
	Hearing impairment	1 (2)
Lactic acidosis severity	Normal	23 (43)
	Mild	22 (42)
	Moderate	7 (13)
	Severe	1 (2)
Myopathies by pathologic features	Normal	10 (19)
	One pathology	23 (43)
	Two pathologies	20 (38)
Biochemical enzyme assay	MRC I complex deficiency	48 (92)
	MRC II complex deficiency	1 (2)
	MRC IV complex deficiency	3 (6)
Syndromic diagnosis	Leigh syndrome	11 (21)
	MELAS	2 (4)
	Non-specific MD	40 (76)

*Data given as total number (percentage) or mean ± SD*.

*MD, mitochondrial disease; MRC, mitochondrial respiratory chain; MELAS, mitochondrial encephalopathy, lactic acidosis, and stroke-like episodes*.

**Table 2:** **Clinical progress of children with mitochondrial disease (*n* = 53)**.

Characteristic		Value
Number of organs involved		1.54 ± 0.93
Organ involvement	CNS only	34 (64)
	CNS + other organs	19 (36)
Severity of epilepsy	No epilepsy	10 (19)
	Drug-responsive epilepsy	18 (33)
	Drug-resistant epilepsy	26 (48)
Diffuse brain atrophy on MRI	Normal	22 (42)
	Mild	26 (49)
	Severe	5 (9)
Clinical progress	Improving	1 (2)
	Static	20 (38)
	Mildly deteriorating	22 (42)
	Severely deteriorating	10 (19)

*Data given as total number (percentage) or mean ± SD*.

*CNS, central nervous system; MRI, magnetic resonance imaging*.

### Effects of Syndromic Diagnosis, Drug-Resistant Epilepsy, Diffuse Brain Atrophy, and Clinical Rating

In patients diagnosed *via* syndromic diagnosis, no significant differences in DQ were observed between patients with Leigh disease and those with non-specific MD (Table 3). Patients with MELAS were excluded from this analysis because the small number of MELAS cases was insufficient for a meaningful statistical analysis. Similarly, no significant difference in DQ was noted between the subgroups of patients with various types of epilepsy severity (Table 3). However, the presence of diffuse brain atrophy was associated with significantly lower DQ levels of development at the pre-first (78.6 ± 20.0 vs 44.3 ± 35.3, *p* = 0.047) and post-second evaluations (33.8 ± 10.0 vs 23.7 ± 13.4, *p* = 0.033), with similar but not statistically significant trends for the rest of the evaluations. Furthermore, DQ was significantly higher in patients with improving or static clinical progress than in patients with mildly deteriorating or severely deteriorating clinical progress at pre-second (68.5 ± 27.1 vs 39.70 ± 29.9, *p* = 0.002) and diagnosis (44.5 ± 22.8 vs 25.6 ± 23.4, *p* = 0.018).

**Table 3:** **Developmental quotient (DQ) for subgroups of children with mitochondrial disease (total, *n* = 53)**.

DQ		Syndromic diagnosis			Drug-resistant epilepsy			Diffuse brain atrophy			Clinical rating		
		Leigh disease	Non-specific	*p*	No	Yes	*p*	No	Yes	*p*	Improving-static	Deteriorating	*p*
Pre-diagnostic evaluation	Pre-first	68.3 ± 35.4 (*n* = 4)	52.5 ± 33.4 (*n* = 14)	0.425	61.5 ± 30.4 (*n* = 7)	52.6 ± 36.3 (*n* = 11)	0.536	78.6 ± 20.0 (*n* = 6)	44.3 ± 35.3 (*n* = 11)	0.047*	66.9 ± 31.8 (*n* = 9)	45.2 ± 33.2 (*n* = 9)	0.177
	Difference Pre-first vs Pre-second	23.6 ± 38.6 (*n* = 4)	12.2 ± 20.4 (*n* = 14)	0.433	27.2 ± 22.2 (*n* = 7)	6.8 ± 23.6 (*n* = 11)	0.151	17.0 ± 21.9 (*n* = 6)	14.3 ± 27.9 (*n* = 11)	0.841	7.1 ± 9.24 (*n* = 9)	22.4 ± 32.7 (*n* = 9)	0.207
	Pre-second	55.8 ± 25.9 (*n* = 11)	49.1 ± 33.7 (*n* = 35)	0.545	54.2 ± 31.0 (*n* = 23)	48.6 ± 33.1 (*n* = 24)	0.632	63.9 ± 33.2 (*n* = 16)	44.8 ± 30.1 (*n* = 30)	0.055	68.5 ± 27.1 (*n* = 19)	39.7 ± 29.9 (*n* = 28)	0.002**
	Difference Pre-second vs diagnosis	20.7 ± 16.2 (*n* = 8)	16.7 ± 24.3 (*n* = 24)	0.676	22.3 ± 18.4 (*n* = 13)	16.4 ± 25.3 (*n* = 20)	0.250	20.1 ± 24.8 (*n* = 9)	18.6 ± 22.8 (*n* = 23)	0.878	18.4 ± 18.0 (*n* = 13)	18.9 ± 25.7 (*n* = 20)	0.951

Diagnostic evaluation	Diagnosis	31.3 ± 26.3 (*n* = 8)	32.5 ± 25.2 (*n* = 29)	0.903	36.0 ± 23.6 (*n* = 17)	30.4 ± 25.7 (*n* = 22)	0.392	43.1 ± 19.3 (*n* = 14)	26.7 ± 26.3 (*n* = 24)	0.051	44.5 ± 22.8 (*n* = 15)	25.6 ± 23.3 (*n* = 24)	0.018*

Post-diagnostic evaluation	Difference Diagnosis vs post-first	11.8 ± 7.5 (*n* = 5)	5.4 ± 8.2 (*n* = 14)	0.147	8.8 ± 9.1 (*n* = 8)	4.1 ± 10.0 (*n* = 13)	0.595	5.4 ± 15.9 (*n* = 5)	5.7 ± 7.9 (*n* = 15)	0.952	8.7 ± 9.9 (*n* = 10)	3.3 ± 9.4 (*n* = 11)	0.214
	Post-first	30.2 ± 21.3 (*n* = 5)	25.4 ± 25.3 (*n* = 14)	0.709	27.4 ± 24.7 (*n* = 8)	29.6 ± 25.8 (*n* = 13)	0.697	44.0 ± 23.0 (*n* = 5)	23.9 ± 24.9 (*n* = 15)	0.130	38.0 ± 22.6 (*n* = 10)	20.4 ± 24.6 (*n* = 11)	0.106
	Difference Post-first vs post-second	5.5 ± 5.0 (*n* = 4)	11.3 ± 18.6 (*n* = 10)	0.559	2.8 ± 4.2 (*n* = 4)	12.1 ± 17.3 (*n* = 11)	0.343	6.0 ± 6.7 (*n* = 4)	12.4 ± 17.9 (*n* = 10)	0.507	16.8 ± 23.2 (*n* = 6)	4.9 ± 3.6 (*n* = 9)	0.267
	Post-second	17.0 ± 18.9 (*n* = 4)	13.3 ± 13.7 (*n* = 10)	0.687	13.6 ± 20.0 (*n* = 4)	19.1 ± 19.2 (*n* = 11)	0.343	33.8 ± 10.0 (*n* = 4)	23.7 ± 13.4 (*n* = 10)	0.033*	25.33 ± 15.9 (*n* = 6)	12.43 ± 19.8 (*n* = 9)	0.106

Data given as total number or mean ± SD. Non-parametric Mann–Whitney analysis with Bonferroni correction: *p < 0.05, **p < 0.01. Pre-first, first pre-diagnostic evaluation; pre-second, second pre-diagnostic evaluation; post-first, first post-diagnostic evaluation; post-second, second post-diagnostic evaluation.

### Effect of Age at First Symptom Onset and Lead Time to Diagnosis

Age at the first symptom onset showed positive correlation with the level of developmental function at post-first (*r* = 0.438, *p* = 0.047) and post-second (*r* = 0.619, *p* = 0.014). Lead time to diagnosis was negatively associated with DQ at all-time points, but this result did not reach statistical significance (Table 4).

**Table 4:** **Correlation between the developmental quotient (DQ) and age at symptom onset, age at diagnosis, and lead time to diagnosis**.

DQ		Age at symptom onset	Age at diagnosis	Lead time to diagnosis
Pre-diagnostic evaluation	Pre-first	0.048	−0.283	−0.305
	Pre-second	0.279	−0.104	−0.237
Diagnostic evaluation	Diagnosis	0.247	−0.086	−0.266
Post-diagnostic evaluation	Post-first	0.438*	0.225	−0.141
	Post-second	0.619*	0.278	−0.188

Significant correlation: *p < 0.05.

*Pre-first, first pre-diagnostic evaluation; pre-second, second pre-diagnostic evaluation; post-first, first post-diagnostic evaluation; post-second, second post-diagnostic evaluation*.

### Long-term Developmental Trends in Children with MDs

**Figure 1:**
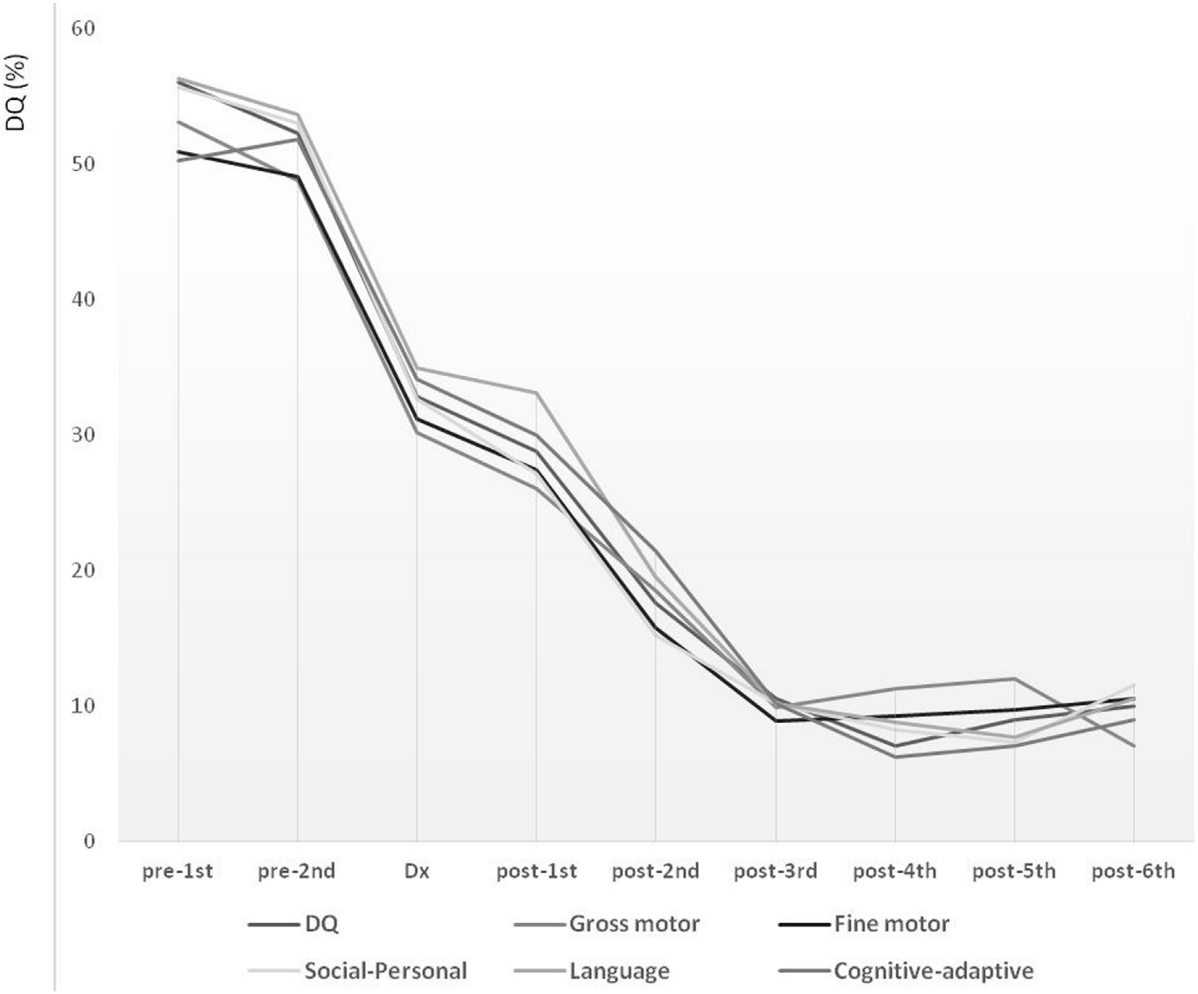
**Long-term developmental trends in children with mitochondrial disease evaluated at nine time points over the course of 7.7 years**. Development level is expressed in terms of developmental quotient (DQ). Pre-diagnostic developmental evaluations were performed twice (pre-first and pre-second), followed by a one-time diagnostic evaluation (Dx) and six post-diagnostic developmental evaluations (post-first, post-second, post-third, post-fourth, post-fifth, and post-sixth).

Long-term follow-up over the course of 7.70 years (from pre-first to post-sixth) showed declining trends over all studied periods (Figure 1). During the lead time to diagnosis, the slope of DQ declined significantly, reflecting a faster deterioration compared with that noted between diagnosis and post-first. The DQ declined steeply also between post-first and post-third. These declining patterns were comparable for the overall development, as well as for the five development subdomains describing gross motor, fine motor, social-personal, language, and cognitive-adaptive skills.

The long-term follow-up over the course of 5.45 years (from pre-first to post-second) showed consistent patterns of developmental deterioration (Table 5). Significant developmental deteriorations were noted for the overall DQ between pre-first and pre-second (difference, 14.7 ± 24.6, *p* = 0.021), as well as between pre-second and diagnosis (difference, 18.7 ± 22.7, *p* < 0.001). Similar patterns of developmental deteriorations were observed in terms of gross motor (difference: pre-first to pre-second, 10.7 ± 20.6, *p* = 0.042; pre-second to diagnosis, 13.7 ± 25.4, *p* = 0.004) and fine motor skills (difference: pre-first to pre-second, 12.6 ± 20.7, *p* = 0.020; pre-second to diagnosis, 17.9 ± 26.3, *p* < 0.001). In addition, evident developmental deteriorations were observed in terms of social–personal (difference, 20.2 ± 31.6, *p* < 0.001), language (difference, 21.5 ± 27.8, *p* < 0.001), and cognitive-adaptive skills (difference, 20.7 ± 27.9, *p* < 0.001), but only between pre-second and diagnosis. In contrast, no significant differences were found for the post-diagnostic evaluations (i.e., between diagnosis and post-first, and between post-first and post-second).

**Table 5:** **Evolution of DQ from the first pre-diagnostic evaluation to the second post-diagnostic evaluation, in terms of various skills of children with mitochondrial diseases (total, *n* = 53)**.

DQ	Subtrahend vs minuend	*n*	Subtrahend	Minuend	Difference	*p*
Total	Pre-first vs pre-second	18	56.0 ± 33.5	41.3 ± 29.4	14.7 ± 24.6	0.021*
	Pre-second vs diagnosis	33	48.8 ± 31.3	30.1 ± 25.0	18.7 ± 22.7	<0.001***
	Diagnosis vs post-first	21	34.6 ± 26.2	28.8 ± 24.8	5.9 ± 9.8	0.013
	Post-first vs post-second	15	27.3 ± 25.5	17.6 ± 18.9	9.7 ± 15.3	0.029

Gross motor	Pre-first vs pre-second	18	53.1 ± 32.9	42.4 ± 31.4	10.7 ± 20.6	0.042*
	Pre-second vs diagnosis	33	42.4 ± 32.8	28.7 ± 23.4	13.7 ± 25.4	0.004**
	Diagnosis vs post-first	21	30.1 ± 22.1	26.0 ± 24.3	4.1 ± 14.7	0.223
	Post-first vs post-second	15	25.7 ± 24.8	18.5 ± 18.2	7.1 ± 12.9	0.051

Fine motor	Pre-first vs pre-second	18	50.9 ± 35.8	38.3 ± 27.6	12.6 ± 20.7	0.020*
	Pre-second vs diagnosis	33	45.1 ± 34.1	27.2 ± 28.3	17.9 ± 26.3	<0.001***
	Diagnosis vs post-first	21	32.7 ± 33.1	27.4 ± 28.0	5.2 ± 18.6	0.209
	Post-first vs post-second	15	25.3 ± 27.8	15.7 ± 19.9	9.5 ± 18.1	0.062

Social–personal	Pre-first vs pre-second	18	55.7 ± 39.1	40.2 ± 35.0	15.5 ± 31.6	0.053
	Pre-second vs diagnosis	33	51.9 ± 35.9	31.6 ± 27.7	20.2 ± 31.6	<0.001***
	Diagnosis vs post-first	21	34.9 ± 30.0	27.2 ± 24.3	7.7 ± 21.1	0.110
	Post-first vs post-second	15	25.4 ± 23.6	15.2 ± 14.5	10.1 ± 21.6	0.104

Language	Pre-first vs pre-second	18	56.4 ± 37.8	45.4 ± 31.7	10.9 ± 22.7	0.057
	Pre-second vs diagnosis	33	52.6 ± 33.9	31.0 ± 27.6	21.5 ± 27.8	<0.001***
	Diagnosis vs post-first	21	37.8 ± 30.2	33.1 ± 29.4	4.6 ± 10.7	0.064
	Post-first vs post-second	15	32.8 ± 31.8	19.6 ± 23.4	13.2 ± 18.6	0.020

Cognitive-adaptive	Pre-first vs pre-second	18	50.3 ± 39.3	38.6 ± 35.3	11.7 ± 31.3	0.131
	Pre-second vs diagnosis	33	51.6 ± 37.1	30.8 ± 29.0	20.7 ± 27.9	<0.001***
	Diagnosis vs post-first	21	35.8 ± 33.1	30.0 ± 29.2	5.8 ± 14.5	0.081
	Post-first vs post-second	15	30.7 ± 31.1	21.4 ± 24.6	9.2 ± 16.1	0.051

Data given as total number or mean ± SD. Differences assessed via the paired t-test with Bonferroni correction: *p < 0.05, **p < 0.01, ***p < 0.001. Pre-first, first pre-Dx; pre-second, second pre-Dx; post-first, first post-diagnostic state; post-second, second post-diagnostic state; DQ, developmental quotient; Dx, diagnostic evaluation.

### Long-term Development Before and After Diagnosis

Repeated measures ANOVA revealed a significant difference in the rate of developmental decline for the pre- and post-diagnosis periods (*p* < 0.001). The subsequent *post hoc* test indicated that these differences were significant for all types of skills.

Repeated measures analysis showed a persistent pattern of developmental deterioration between the pre-diagnostic and post-diagnostic periods for overall development [difference, 16.1; 95% confidence interval (CI), 6.3–25.8, *p* = 0.001], as well as gross motor (difference, 12.8; 95% CI, 1.8–23.8, *p* = 0.020), fine motor (difference, 16.2; 95% CI, 2.2–30.2, *p* = 0.020), social–personal (difference, 16.3; 95% CI, 0.8–31.8, *p* = 0.037), language (difference, 20.7; 95% CI, 6.1–35.2, *p* = 0.005), and cognitive-adaptive (difference, 15.3; 95% CI, 1.0–29.5, *p* = 0.033) skills (Table 6). The overall DQ showed a decline from the pre-diagnostic through the post-diagnostic periods, suggesting an overall deterioration.

**Table 6:** **Repeated measures ANOVA for long-term development of various skills in children with mitochondrial diseases (*n* = 18)**.

DQ	Difference	*p*	Difference (95% confidence interval)	*Post hoc*
Total		<0.001		
	Pre-Dx		16.1 (6.3–25.8)	0.001**
	Dx-post		7.7 (2.4–13.0)	0.004**

Gross motor		<0.001		
	Pre-Dx		12.8 (1.8–23.8)	0.020*
	Dx-post		6.8 (0.6–14.3)	0.080

Fine motor		<0.001		
	Pre-Dx		16.2 (2.2–30.2)	0.020*
	Dx-post		7.7 (1.0–16.4)	0.095

Social–personal		<0.001		
	Pre-Dx		16.3 (0.8–31.8)	0.037*
	Dx-post		11.4 (0.7–23.5)	0.071

Language		<0.001		
	Pre-Dx		20.7 (6.1–35.2)	0.005**
	Dx-post		4.3 (2.8–11.5)	0.381

Cognitive-adaptive		0.001		
	Pre-Dx		15.3 (1.0–29.5)	0.033*
	Dx-post		6.4 (2.7–15.6)	0.240

General linear model, repeated measures with Bonferroni adjustment: *p < 0.05, **p < 0.01. DQ, developmental quotient; pre, pre-diagnostic evaluation; post, post-diagnostic evaluation; Dx, diagnostic evaluation.

## Discussion

In the present study, the global developmental trends were evaluated over a follow-up period of 7.70 years at nine time points and showed a decline over all periods. However, even with consistently declining patterns, the characteristics of developmental deterioration were disparate. The overall developmental decline pattern could be classified into five phases: (1) pre-diagnostic initial decline phase; (2) pre-diagnostic accelerated decline phase; (3) post-diagnostic alleviated phase; (4) post-diagnostic reaccelerated decline phase; and (5) post-diagnostic stagnant phase.

Considering the pre-diagnostic phase as lead time, the DQ declined continuously, with the initial decline starting from the first onset of symptoms, followed by an accelerated deteriorating phase until diagnosis. During the initial period from diagnosis to the first post-diagnostic evaluation (~1 year), an impeded phase of developmental decline was noted, which appeared alleviated compared with the decline noted in previous phases. This was followed by an accelerated period of decline for ~2 years. Finally, ~3 years after the diagnosis, the decline reached a stagnant phase. In this phase, the developmental deterioration appeared to be reaching the lowest and terminal level of 10% of the DQ. These declining patterns were similar for the overall development and all types of skills, including the gross motor, fine motor, social–personal, language, and cognitive-adaptive. However, a more detailed analysis revealed that the delayed development in gross and fine motor skills exhibited more significant deterioration even in the pre-diagnostic initial decline phase.

In terms of the lead time, the developmental decline accelerated from the first onset of symptoms to the time of confirmation of diagnosis. In contrast, right after the diagnosis, a post-diagnostic alleviated phase was manifested. These facets of the developmental course highlight the importance of shortening the lead time as well as of early detection of the first significant symptom. A shorter lead time is important in achieving a longer developmentally alleviated phase or a prolonged period before reaching the stagnant phase and the lowest and terminal level of development.

As developmental delay is one of the first common symptoms in children with MD (16), vigilance and careful observation of any motor delay in children are integral to early diagnosis. With more structural attention and information, motor delays might be easier to detect by caregivers because of their comparatively evident developmental milestones in earlier childhood compared with language, cognitive, or social development (7). In addition, any motor delay is usually enough for a child to be referred to professionals, and additional signs of any developmental delays in the language, social, and cognitive domains should be further evaluated for etiological diagnosis. Hence, early detection of motor delay and other developmental signs of delay is important in children with MD to shorten the lead time.

Furthermore, the effects of diagnosis and intervention should not be overlooked in children with MD. Such a standardized screening tool to detect MDs early at patients’ routine visits to the pediatricians would be ideal. However, making the correct diagnosis is exceedingly problematic because of the heterogeneous features of this disorder (17, 18). MD may present at any age, with a spectrum of symptoms and signs, to several medical specialties. And there are no standard guidelines for the investigation of MD (2, 3). Diagnostic difficulty results not only from the wide spectrum of symptoms and signs that an individual patient may have but also from the absence of a reliable screening or diagnostic biomarker that is both sensitive and specific in all cases of MD (4). Discussion with a clinician who has expertise in MD is advised in order to guide investigation, in particular with respect to laboratory facilities and handling of specimens (2). Therefore, it might not be easy to adopt a standardized screening tool to detect them early in MD.

Confirming the diagnosis of MD is important to both the parents and physician, because confirmation of diagnosis could relieve the parents’ anxiety and helplessness caused by the uncertainty of an undiagnosed condition and encourage them to seek various therapies for their children. For physicians, confirming the diagnosis of MD is fundamental in planning therapeutic intervention (17, 18).

The appearance of a post-diagnostic alleviated phase in this study is notable and possibly even inspiring to physicians who have only been using symptomatic or supportive treatment for MD. The possibility of a positive effect of the treatment on patients’ development, including mitochondrial cocktails, such as coenzyme Q, carnitine, and various vitamins, which were provided to the children with MD in this study, should not be dismissed. Notably, the children with MD in this study had been taking a mitochondrial cocktail immediately after receiving a confirmed diagnosis. Accordingly, the necessity of an early diagnosis, consecutive symptomatic treatment, general supportive care, and early administration of possible medicine to shorten the lead time to diagnosis and alleviate the patients’ developmental regression is suggested.

We found that diffuse brain atrophy, the clinical rating provided by the physician, and the age at first symptom onset significantly affected the developmental level and decline. However, no significant effect was noted for syndromic diagnosis and epilepsy severity, suggesting that these aspects may not directly reflect the developmental condition of the patients. Because of the heterogeneous symptoms of MDs (14), syndromic diagnosis based on a combination of particular symptoms might not be strongly associated with the developmental function of children with MDs. Additionally, epilepsy-related factors are not the main factors that influence developmental function in such patients, but are nonetheless important manifestations of MDs (14). Instead, diffuse brain atrophy might largely account for the developmental level as a measure of brain function with structural change (16, 19). In particular, all pediatric patients included in the present study presented with central nervous system involvement. In addition, more than half had diffuse brain atrophy, which was associated with lower developmental level at all-time points and over all periods considered, from the early stage of the pre-diagnostic period through the lowest, stagnant, and terminal phase. The concept of clinical ratings (20) provided by the treating physician could be regarded as a good predictor of long-term developmental prognosis because it reflects the characteristics of the patient’s general state and multiorgan involvement.

This study has several limitations. This is a preliminary study with a small number of patients, and the overall cohort was not followed up consistently since we reviewed the data retrospectively. Moreover, in spite of the advantage of other developmental scales such as Bayley scale of Infants Development, KICDT was used as the most frequently and repeatedly used measure in outpatient or inpatient clinical settings in the current study. It still has a strength as a possible long-term and repeatedly useful screening tool, however, other developmental measures might be able to give more reliable information about the patients’ developmental function. In addition, patients’ anthropometric values were not included in the current study due to the small number of patients as a preliminary study. Despite such limitations, the present study addresses the lack of data regarding the long-term developmental trajectory and clinical course of pediatric patients with MDs, as such diseases are characterized by diverse and heterogeneous feature manifestations (7, 20, 21). Even a rough estimate of the long-term developmental trajectory of children with MD could provide important information for diagnosis and development of possible intervention plans. In future study, we hope that we could overcome this limitation on the heterogeneity of MD with a larger number of patients and include prospective data as well based on the understanding of developmental course of pediatric MD from current preliminary data.

Despite the study limitations, our findings highlight the importance of DQ in the prediction and rating of the long-term functional course of children with MD. There has been no other established predictor in terms of patient’s functional measure in pediatric MD because of its difficulties of expecting the clinical course. In the perspective of this limitation, development quotient might be a candidate as a predictor or a measure in pediatric MD by giving the functional level of patients, which is a very meaningful value in pediatrics and could be obtained even from pediatric MD patients with much delayed developmental level. Our observations suggest that, upon confirmation of the diagnosis by the physician, measures, such as symptomatic treatment, general supportive care, and early administration of medicine, are encouraged to shorten the lead time to diagnosis and thereby minimize the patients’ developmental regression.

## Ethics Statement

All procedures were approved by the institutional review board of Gangnam Severance Hospital in Seoul, Korea.

## Author Contributions

SE conceptualized and designed the study, carried out the data analysis, drafted the initial manuscript, reviewed and revised the manuscript, and approved the final manuscript as submitted. Y-ML conceptualized and designed the study, critically reviewed and revised the manuscript, and approved the final manuscript as submitted. Both listed authors meet the appropriate authorship criteria. No one who qualifies for authorship has been omitted from the list. Contributors and their funding sources have been properly acknowledged, and authors and contributors have approved the acknowledgment of their contributions.

## Conflict of Interest Statement

The authors declare that they have no financial relationships relevant to this article. The funders had no role in study design, data collection, data analysis, interpretation of results, manuscript preparation, or decision to submit the article for publication.
